# The transcriptional landscape of lncRNAs reveals the oncogenic function of *LINC00511* in ER-negative breast cancer

**DOI:** 10.1038/s41419-019-1835-3

**Published:** 2019-08-08

**Authors:** Jian Zhang, Shiyao Sui, Hao Wu, Jinfeng Zhang, Xingda Zhang, Shouping Xu, Da Pang

**Affiliations:** 1 Department of breast surgery, Harbin Medical University Cancer Hospital, 150 Haping Road, 150081 Harbin, China; 2 Heilongjiang Academy of Medical Sciences, 157 Baojian Road, 150086 Harbin, China

**Keywords:** Breast cancer, Long non-coding RNAs

## Abstract

Advances in the molecular characteristics of cancers have facilitated the classification system from morphology to molecular characteristic-based subtypes. Cancer profiling has expanded in its focus from protein-coding genes to noncoding RNAs, with advances in the depth and quality of transcriptome sequencing. Here, we examined the profiles of long noncoding RNAs (lncRNAs) according to breast cancer subtype categories in The Cancer Genome Atlas (TCGA) database to identify a cohort of breast cancer- and oestrogen receptor (ER)-negative-associated lncRNAs. According to the prioritization of variation in ER-negative-associated lncRNAs, we identified and investigated the role of *LINC00511* in breast cancer. We determined that high *LINC00511* expression was an unfavourable prognostic factor for patients with breast cancer. Furthermore, *LINC00511* promoted tumour growth by accelerating the G1/S transition and inhibiting apoptosis. At the transcriptional level, ER deficiency directly affected the expression of *LINC00511* activated by transcription factor AP-2 (TFAP-2) in breast cancer cells. Moreover, mechanistic investigations demonstrated that ER-negative-associated *LINC00511* interacted with enhancer of zeste homologue 2 (EZH2, the catalytic subunit of polycomb repressive complex 2, PRC2) and recruited PRC2 to mediate histone methylation, contributing to the repression of *CDKN1B* in the nucleus. This process resulted in altered ER-negative breast cancer cell biology. By highlighting the oncogenic function of *LINC00511*, we revealed the role of lncRNAs in regulating the network of cell cycle control in ER-negative breast cancer and suggested the exploitation of *LINC00511* as an anticancer therapy in the future.

## Introduction

Breast cancer is one of the most commonly diagnosed cancers and the leading cause of death in women worldwide^1^. With advances in the recognition of the molecular characteristics of cancers, breast cancer has been categorized into four major subtypes: luminal A, luminal B, human epidermal growth factor receptor 2 (HER2)-positive and basal-like^2,3^. Among the major molecular characteristics of breast cancer, oestrogen receptor (ER) is crucial to the classification of breast cancer subtypes and tailored individualized therapy^4,5^. Aberrant ER transcriptional activity is involved in the endocrine response and cell cycle progression^6,7^. Patients with ER-negative breast cancer tend to have a poorer prognosis than those with ER-positive breast cancer^8^. However, the network of underlying regulatory mechanisms in the initiation and progression of ER-negative breast cancer remains poorly understood.

Long noncoding RNAs (lncRNAs) are heterogeneous categories of transcripts more than 200 base pairs in length. Because they lack the ability to translate into proteins, they were once considered “transcriptional noise”^9,10^. In recent years, the “transcriptional noise” has no longer been considered irrelevant, and lncRNAs were confirmed to be involved in multiple pathological conditions such as carcinogenesis and cancer progression^11–13^. In our previous study, we reported that the lncRNA *EGOT*, a downregulated lncRNA in cancer tissues, enhanced cellular autophagosome accumulation and sensitized cells to paclitaxel in breast cancer^9,14^. With advances in scientific research, the old, traditional dogmas of prominent RNA functions were overthrown and a supplement or modification to traditional understanding was gradually established^10,13,15^.

Our cognition of cancer is an evolving process. Douglas Hanahan and Robert A. Weinberg provided an excellent overview summarizing ten hallmarks of cancer in 2011^16^. Among these hallmarks, epigenetic regulation, identified as a heritable variation, plays a significant role in tumourigenesis. Aberrant epigenetic regulation contributes to the chaos of gene expression, chromatin organization, cell differentiation, etc^17,18^. Some lncRNAs have been reported to serve as epigenetic regulators influencing the expression of target genes, such as *MALAT1*, *PVT1* and *HOTAIR*^18–21^. Moreover, sustained cell proliferation, which ensures the continuity of heritable variations in cancer, such as genetic modifications, was identified as another important hallmark of cancer. Uncontrolled cell proliferation results from defects in the control of the cell cycle, particularly at the G1/S transition^22^. To date, some lncRNAs have been confirmed to regulate the G1/S transition to affect tumour growth^22–24^. Often, the hallmarks of cancer are interwoven rather than isolated, similar to the regulatory mechanisms of lncRNAs in cancers. Some lncRNAs, such as *SNHG1*, have been reported to epigenetically repress the expression of cyclin-dependent kinase inhibitors (CDKIs) to promote the cell cycle^25^.

Here, we repurposed and integrated multi-RNA-sequencing (RNA-seq) analyses of lncRNA expression profiles among entries in the breast tissue cohort from The Cancer Genome Atlas (TCGA) database to identify the lncRNAs that are enriched in both breast cancer (cancer versus normal) and ER-negative breast cancer (ER-negative versus ER-positive)^11,13^. We identified and investigated the role of *LINC00511* in breast cancer according to the prioritization of variation in ER-negative-associated lncRNAs. In previous studies, *LINC00511* was reported to exert an oncogenic function in many cancers, such as breast cancer, non-small cell lung cancer, ovarian cancer and glioma^26–29^. We found that *LINC00511* promoted tumour growth by accelerating the G1/S transition and inhibiting apoptosis in breast cancer. We demonstrated that ER deficiency directly affected the expression of *LINC00511* activated by TFAP-2 and identified EZH2, which is involved in histone methylation, as a protein that interacts with *LINC00511* and contributes to the repression of *CDKN1B* in ER-negative breast cancer. We aimed to provide detailed knowledge of *LINC00511* in breast cancer progression. By integrating these data with the TCGA clinical data, we aimed to identify and validate the carcinogenic mechanism of *LINC00511* in ER-negative breast cancer. In the future, it may be exploited for anticancer therapy.

## Results

### Identification of breast cancer- and ER-negative-associated lncRNAs

**Fig. 1:**
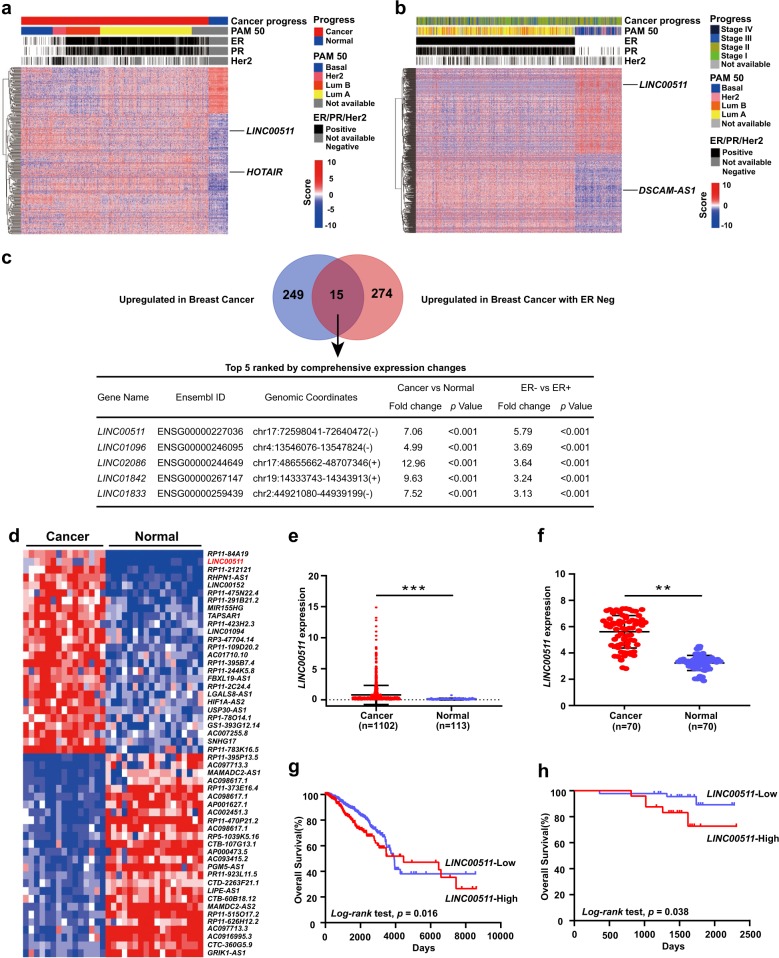
Identification of breast cancer- and ER-negative-associated lncRNAs. **a** Hierarchical clustering heat map of the differentially expressed lncRNAs in breast cancer tissues and adjacent normal tissues generated from RNA-seq data from the TCGA database. A total of 342 lncRNAs were differentially expressed, with a *p* value < 1E-20 and a fold change >2. Among these lncRNAs, 264 were upregulated in breast cancer tissues, and 78 were upregulated in adjacent normal tissues. The cancer progression, PAM50, ER, PR and HER2 statuses of each sample are shown above the heat map. *HOTAIR* was employed as a positive control. **b** Hierarchical clustering heat map of the differentially expressed lncRNAs in ER-positive and ER-negative breast cancer tissues generated from RNA-seq data from the TCGA database. A total of 551 lncRNAs were differentially expressed, with a *p* value < 1E-4 and a fold change >2. Among these lncRNAs, 262 were upregulated in ER-positive breast cancer tissues, and 289 were upregulated in ER-negative breast cancer tissues. The cancer progression, PAM50, ER, PR and HER2 statuses of each sample are shown above the heat map. *DSCAM-AS1* was employed as a positive control. **c** Venn diagram depiction of the intersection of the lncRNAs upregulated in both breast cancer and ER-negative breast cancer tissues (264 versus 289, respectively). The top five lncRNAs are listed according to the prioritization of variation in ER-negative-associated lncRNAs. **d** Heat map of the differentially expressed lncRNAs in breast cancer tissues, adjacent normal tissues and non-breast cancer patient tissues generated from RNA-seq data of the whole-transcriptome sequencing of 33 breast cancer specimens (RNA-seq data of this study has been deposited into the NCBI GEO database under accession number GSE71651). **e** Scatter diagram depiction of *LINC00511* expression in breast cancer tissues and adjacent normal tissues generated from the TCGA database. **f** qRT-PCR analysis of *LINC00511* expression in 70 pairs of breast cancer and adjacent normal tissues. **g** Kaplan–Meier survival analysis of OS in breast cancer patients based on *LINC00511* expression generated from the TCGA database (*n* = 1086, high = 379, low = 707, *p* < 0.05). **h** Kaplan–Meier survival analysis of OS in breast cancer patients based on *LINC00511* expression in our cohort (*n* = 70, high = 24, low = 46, *p* < 0.05). Data are shown as the mean ± standard deviation (SD). Student’s *t* test was used for the statistical analysis: *p < 0.05; **p < 0.01; ***p < 0.001. Data represent three independent experiments

Utilizing the large-scale cancer genome RNA-seq expression data from the TCGA database, we focused on the potential carcinogenesis of lncRNAs that were differentially expressed in breast cancer tissues compared with adjacent normal tissues. We identified 264 upregulated lncRNAs in breast cancer tissues (Fig. 1a). *HOTAIR*, a well-known lncRNA that promotes breast cancer aggression, was included^30^. We also identified differentially expressed lncRNAs in ER-positive breast cancer tissues compared with ER-negative tissues within the lncRNA expression profiles in cancer tissues. We identified 289 upregulated lncRNAs in ER-negative breast cancer tissues (Fig. 1b). Among these lncRNAs, *DSCAM-AS1* was affirmed to be responsible for the oncogenicity in ER-positive breast cancer^11^. Because of a poor prognosis encountered by patients with ER-negative breast cancer (relative to patients with ER-positive breast cancer), we investigated the potential oncogenic function of ER-negative-associated lncRNAs that were upregulated in both breast cancer (cancer versus normal) (Fig. 1a) and ER-negative breast cancer (ER-negative versus ER-positive) (Fig. 1b). We examined the intersection of these two lncRNA profiles, and 15 lncRNAs were upregulated in both the cancer versus normal analysis and in the ER-negative versus ER-positive analysis, as shown in the Venn diagram (Fig. 1c and Table S1). According to the prioritization of variation in ER-negative-associated lncRNAs, we selected *LINC00511* for further investigation.

We also examined the protein-coding potential of *LINC00511* through the CPAT database (http://lilab.research.bcm.edu/cpat/) and the CPC database (http://cpc.cbi.pku.edu.cn/) (Fig. S1a, b). Moreover, an RNA pull-down assay showed no interaction/binding between *LINC00511* and ribosomal protein LP0, a component of the 60S ribosomal subunit (Fig. S1c)^31,32^. These data suggest that *LINC00511* has no protein-coding potential.

### LINC00511 expression is increased in breast cancer and correlates with a poor prognosis

The whole-transcriptome sequencing of 33 breast cancer specimens, including breast cancer tissue (*N* = 15), adjacent normal tissue (*N* = 15) and non-breast cancer patient tissue (*N* = 3), showed that *LINC00511* was highly expressed in cancer tissues compared with adjacent normal tissues (Fig. 1d). The expression of *LINC00511* was further validated by analysing 70 pairs of breast cancer and adjacent normal tissues from the Harbin Medical University Cancer Centre (HMUCC) and RNA-seq data from the TCGA database (Fig. 1e, f). To explore the relationship between *LINC00511* expression and the prognosis of breast cancer patients, we performed a Kaplan–Meier analysis and a log-rank test to assess the effects of *LINC00511* expression and clinical outcomes on overall survival in the TCGA database and 70 paired breast cancer tissues, respectively. As shown in Fig. 1g, h, high *LINC00511* expression indicated a remarkably poorer prognosis than low *LINC00511* expression did in patients. Next, we examined the correlation of *LINC00511* expression with patients’ clinicopathological characteristics in breast cancer. *LINC00511* expression was positively correlated with tumour size, ER, progesterone receptor (PR), Ki-67 and p53 status (*p* < 0.05). No significant association was found between *LINC00511* expression and age, tumour-node-metastasis (TNM) stages, histological grade, lymph-node-metastasis (LNM) or HER2 status (Table 1).

**Table 1:** Correlation between *LINC00511* expression and clinicopathological characteristics of breast cancer patients

Clinical parameter test *p*-value	No. cases (*n* = 70)	*LINC00511* expression		Chi-squared
		High cases	Low cases	
Age (years)				0.75
≤50	39	14	25	
>50	31	10	21	
Tumour size (cm)				0.03
≤2	33	7	26	
>2	37	17	20	
TNM stages				0.08
I–II	65	20	45	
III–IV	5	4	1	
Histological grade				0.77
I–II	51	18	33	
III	19	6	13	
LNM				0.74
Positive	36	13	23	
Negative	34	11	23	
ER status				<0.01
Positive	19	1	18	
Negative	51	23	28	
PR status				0.04
Positive	14	1	13	
Negative	56	23	33	
Her2 status				0.06
Positive	37	9	28	
Negative	33	15	18	
Ki-67 status				0.02
≤14%	54	23	31	
>14%	16	1	15	
P53 status				<0.01
Positive	29	16	13	
Negative	41	8	33	

*LNM* lymph node metastasis

### Characteristics of LINC00511 in ER-negative breast cancer

**Fig. 2:**
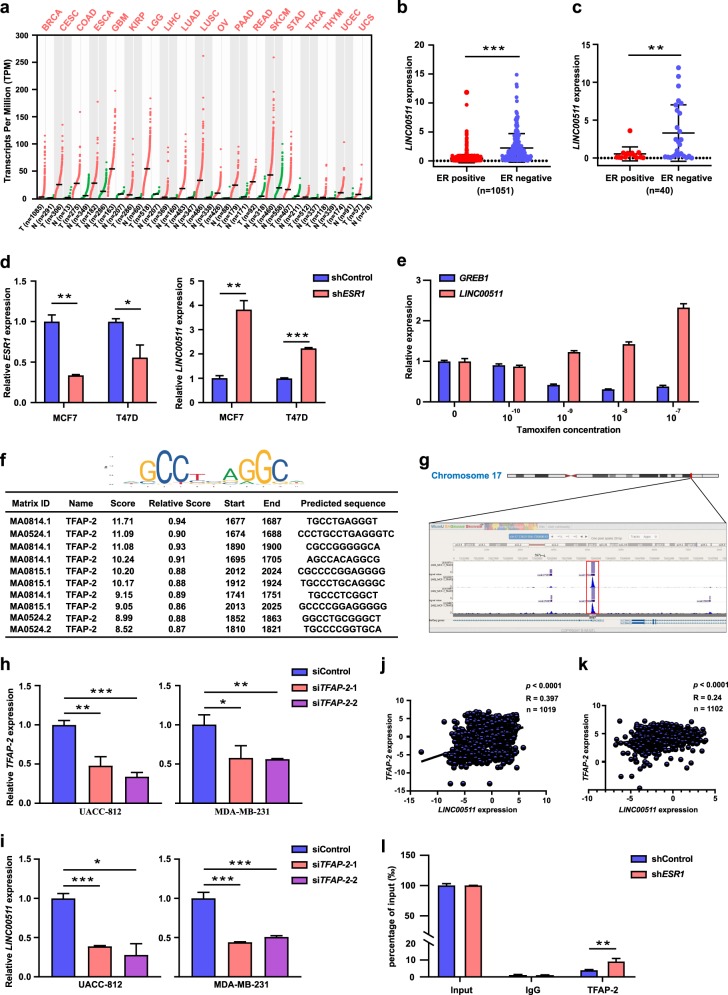
Characteristics of *LINC00511* in ER-negative breast cancer. **a** Scatter diagram depiction of *LINC00511* expression in multiple cancer tissues and adjacent normal tissues generated from the GEPIA database. **b** Scatter diagram depiction of *LINC00511* expression in ER-positive and ER-negative breast cancer tissues generated from the TCGA database. **c** Scatter diagram depiction of *LINC00511* expression in ER-positive and ER-negative breast cancer cells generated from the CCLE database. **d** qRT-PCR analysis of *LINC00511* expression following the knockdown of *ESR1* expression in MCF7 and T47D cell lines. **e** qRT-PCR analysis of *LINC00511* expression in MCF7 cells treated with different doses of tamoxifen. **f** The upper corner of the picture showed the TFAP-2 binding motif, and the lower table showed the prediction of TFAP-2 binding sites within the promoter region of *LINC00511* provided by the JASPAR database. **g** Analysis of TFAP-2 ChIP-seq data from MCF7 cells at the *LINC00511* promoter locus generated from data from the ENCODE and Cistrome Data Browser databases. **h** qRT-PCR analysis of the knockdown efficiency of *TFAP-2* expression in UACC-812 and MDA-MB-231 cells. **i** qRT-PCR analysis of *LINC00511* expression in UACC-812 and MDA-MB-231 cell lines following the knockdown of *TFAP-2* expression. **j** Scatter diagram analysis of the correlation between *TFAP-2* and *LINC00511* generated from the CCLE database. **k** Scatter diagram analysis of the correlation between *TFAP-2* and *LINC00511* generated from the TCGA database. **l** ChIP and qRT-PCR analysis of TFAP-2 occupancy at the *LINC00511* promoter region and the altered occupancy efficiency following the knockdown of *ER* expression in MCF7 cells. IgG was used as a negative control. Enrichment was quantified relative to input controls. Data are shown as the mean ± SD. Student’s *t* test was used for the statistical analysis: *p < 0.05; **p < 0.01; ***p < 0.001. Data represent three independent experiments

In addition to breast cancer, *LINC00511* exhibited a highly cancer-specific expression pattern in multiple cancers according to an analysis of the Gene Expression Profiling Interactive Analysis database (http://gepia.cancer-pku.cn/) (Fig. 2a). As shown in Fig. 2b, c, *LINC00511* expression was upregulated in the ER-negative breast cancer cohort from the TCGA database and in ER-negative breast cancer cells from the Cancer Cell Line Encyclopedia (CCLE) database (http://portals.broadinstitute.org/ccle/about). To investigate the relationship between *LINC00511* and ER deficiency, we knocked down *ER* expression in MCF7 and T47D cells and found that *LINC00511* expression was upregulated (Fig. 2d). Furthermore, *LINC00511* expression was induced in MCF7 cells after stimulation with the anti-oestrogen agent tamoxifen, and tamoxifen induced *LINC00511* expression in a dose-dependent manner (Fig. 2e). We deduced that certain regulatory mechanisms involved in *LINC00511* expression may be involved when blocking ER signalling.

We utilized bioinformatics to analyse the potential regulation of *LINC00511* expression. Using the University of California at Santa Cruz (UCSC) database (http://genome.ucsc.edu/cgi-bin/hgGateway), we obtained the location information around the transcriptional start site (TSS, −2000 bases to +200 bases, “−” indicates “upstream of the TSS” and “+” indicates “downstream of the TSS”). Then, we used the JASPAR database (http://jaspar.genereg.net/) to scan this segment and performed a promoter binding analysis. We predicted that *LINC00511* was the potential target gene of TFAP-2 (Fig. 2f). We validated this interaction in the ENCODE database (https://www.encodeproject.org/) and in the Cistrome Data Browser database (http://cistrome.org/db/#/) (Fig. 2g). We ascertained the basic expression levels of *LINC00511* in breast cancer cells through qRT-PCR assays, and UACC-812 and MDA-MB-231 cells, which express higher levels of *LINC00511* than other breast cancer cells, were selected for subsequent investigation (Fig. S2). We knocked down *TFAP-2* expression in UACC-812 and MDA-MB-231 cells and observed that *LINC00511* expression was impaired (Fig. 2h, i). We found that *LINC00511* expression was positively correlated with *TFAP-2* in cancer cells and breast cancer tissues according to the CCLE and TCGA databases (Fig. 2j, k). The occupancy of TFAP-2 at the promoter region of *LINC00511* was also confirmed through chromatin immunoprecipitation (ChIP) assays (Fig. 2l). These results illustrated that TFAP-2 promoted *LINC00511* expression at the transcriptional level. Next, we wanted to determine whether there was a correlation between ER deficiency and TFAP-2 activity in regulating *LINC00511* expression. ChIP assays confirmed that the occupancy efficiency of TFAP-2 at the specific promoter region was enhanced after knocking down *ER* expression in MCF7 cells (Fig. 2l). These data indicated that ER deficiency promoted *LINC00511* expression by enhancing the occupancy efficiency of TFAP-2 at specific promoter regions, resulting in increased transcriptional activity.

### LINC00511 impacts ER-negative breast cancer cell proliferation and apoptosis in vitro

**Fig. 3:**
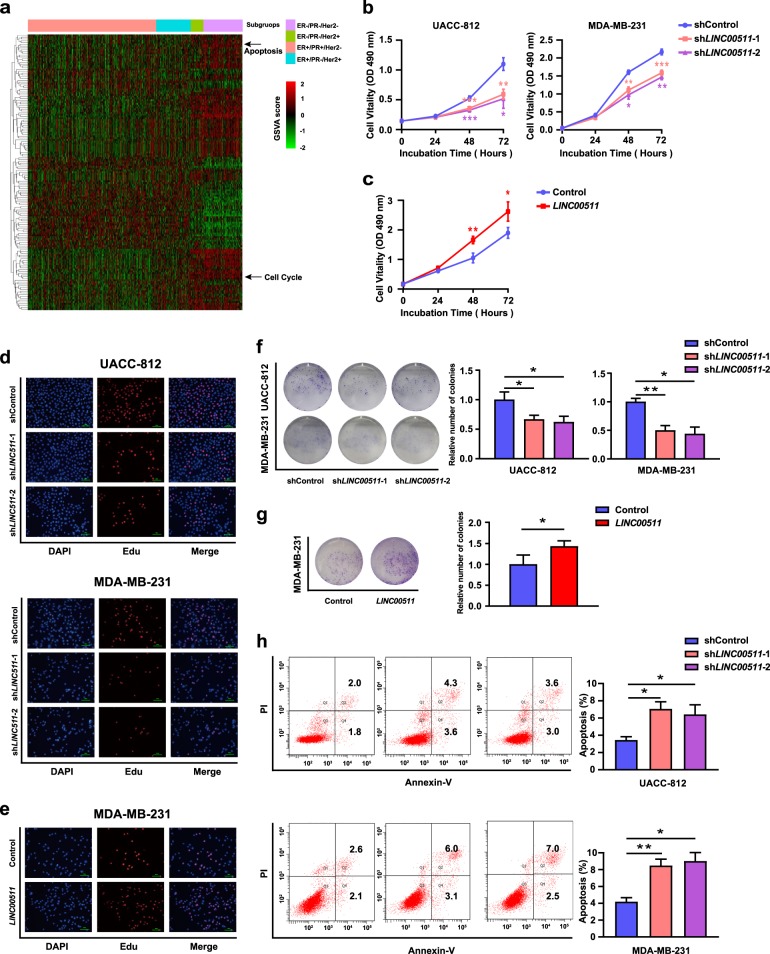
*LINC00511* impacts ER-negative breast cancer cell proliferation and apoptosis in vitro. **a** Cancer gene signature analyses and clustering of GSVA scores for *LINC00511* in breast cancer patients obtained from the TCGA database. **b** CCK-8 analysis of the viability and proliferation of UACC-812 and MDA-MB-231 cells following the knockdown of *LINC00511* expression. **c** CCK-8 analysis of the viability and proliferation of MDA-MB-231 cells following the overexpression of *LINC00511* expression. **d** EdU analysis of the proliferation ability of UACC-812 and MDA-MB-231 cells following the knockdown of *LINC00511* expression. **e** EdU analysis of the proliferation ability of MDA-MB-231 cells following the overexpression of *LINC00511* expression. **f** Analysis of the cell colony formation ability of UACC-812 and MDA-MB-231 cells following the knockdown of *LINC00511* expression. The number of colonies was counted on the 14th day after seeding. **g** Analysis of the cell colony formation ability of MDA-MB-231 cells following the overexpression of *LINC00511* expression. The number of colonies was counted on the 14th day after seeding. **h** Flow cytometry (cell apoptosis) analysis of the apoptosis of UACC-812 and MDA-MB-231 cells following the knockdown of *LINC0051* expression. Data are shown as the mean ± SD. Student’s *t* test was used for the statistical analysis: *p < 0.05; **p < 0.01; ***p < 0.001. Data represent three independent experiments

To gain insight into the functional role of *LINC00511* in ER-negative breast cancer, we performed a gene set variation analysis (GSVA) to determine whether the cancer phenotypes regulated by *LINC00511* were shared across breast cancer patients from the TCGA database^33^ (Fig. 3a). We focused on a set of cancer-specific signatures that are shown in Table S2. Among these cancer-specific signatures, we focused our attention on cell proliferation and cell apoptosis pathways, which were enriched more in ER-negative breast cancer patients than in ER-positive breast cancer patients. *LINC00511* expression was knocked down in UACC-812 and MDA-MB-231 cells and overexpressed in MDA-MB-231 cells, as shown in Fig. S3a–c. Cell Counting Kit-8 assays showed that the knockdown of *LINC00511* expression significantly inhibited the viability and proliferation of UACC-812 and MDA-MB-231 cells relative to shcontrol cells, while the overexpression of *LINC00511* substantially promoted the viability and proliferation of MDA-MB-231 cells (Fig. 3b, c). Ethynyldeoxyuridine (EdU) assays also supported the conclusions in UACC-812 and MDA-MB-231 cells (Fig. 3d, e). The results of colony-forming growth assays revealed that the knockdown of *LINC00511* expression greatly attenuated the colony-forming ability of UACC-812 and MDA-MB-231 cells, but the overexpression of *LINC00511* enhanced the colony-forming ability of MDA-MB-231 cells (Fig. 3f, g). Furthermore, flow cytometry (cell apoptosis) analysis demonstrated a significantly increased proportion of apoptotic cells following the knockdown of *LINC00511* expression (Fig. 3h). Taken together, these results indicated that *LINC00511* promoted ER-negative breast cancer cell growth by affecting cell proliferation and apoptosis.

### LINC00511 accelerates the G1/S transition, in part, by regulating CDKN1B expression

**Fig. 4:**
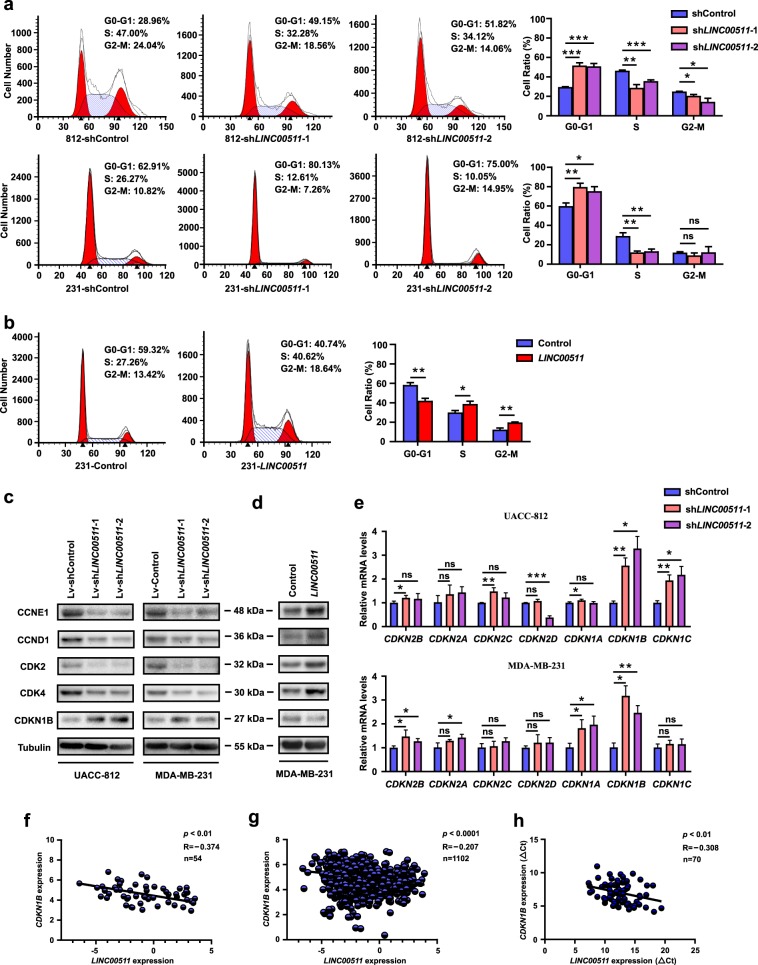
*LINC00511* accelerates the G1/S transition, in part, by regulating *CDKN1B* expression. **a** Flow cytometry (cell cycle distribution) analysis of the proportion of UACC-812 and MDA-MB-231 cells in the G0/G1, S and G2/M phases following the knockdown of *LINC00511* expression. **b** Flow cytometry (cell cycle distribution) analysis of the proportion of MDA-MB-231 cells in the G0/G1, S and G2/M phases following the overexpression of *LINC00511* expression. **c** Western blot analysis of the key cell cycle-related proteins (CCNE1, CCND1, CDK4, CDK2 and CDKN1B) of UACC-812 and MDA-MB-231 cells following the knockdown of *LINC00511* expression. **d** Western blot analysis of the key cell cycle-related proteins (CCNE1, CCND1, CDK4, CDK2 and CDKN1B) of MDA-MB-231 cells following the overexpression of *LINC00511* expression. **e** qRT-PCR analysis of CDKI mRNAs (the INK4 and Cip/Kip families) of UACC-812 and MDA-MB-231 cells following the knockdown of *LINC00511* expression. **f** Scatter diagram analysis of the correlation between *LINC00511* and *CDKN1B* expression obtained from the CCLE database. **g** Scatter diagram analysis of the correlation between *LINC00511* and *CDKN1B* expression generated from the TCGA database. **h** Scatter diagram analysis of the correlation between *LINC00511* and *CDKN1B* expression in 70 breast cancer tissues in our cohort. Data are shown as the mean ± SD. Student’s *t* test was used for the statistical analysis: *p < 0.05; **p < 0.01; ***p < 0.001. Data represent three independent experiments

To investigate the specific regulatory mechanisms of *LINC00511* in promoting cell proliferation, flow cytometry (cell cycle distribution) analysis revealed that the knockdown of *LINC00511* expression significantly decreased the proportion of UACC-812 and MDA-MB-231 cells in S phase. In contrast, the overexpression of *LINC00511* increased the proportion of MDA-MB-231 cells in S phase (Fig. 4a, b). These results indicate that *LINC00511* may play an important role in the G1/S transition of the cell cycle. We also detected key cell cycle-related proteins that play irreplaceable roles in G1 phase. The results revealed that CCNE1, CCND1, CDK4 and CDK2 expression was impaired in UACC-812 and MDA-MB-231 cells following the knockdown of *LINC00511* expression, and the overexpression of *LINC00511* increased the expression of these proteins in MDA-MB-231 cells (Fig. 4c, d).

CDKIs, which are classified into the INK4 and Cip/Kip families, act as brakes to halt cell cycle progression^34^. We suspected that *LINC00511* played a role through CDKIs. qRT-PCR assays were performed to examine the correlation between *LINC00511* and CDKI mRNAs. *CDKN1B* was the only mRNA that was increased in both UACC-812 and MDA-MB-231 cells following the knockdown of *LINC00511* expression (Fig. 4e). Furthermore, the observation was validated at the protein level in UACC-812 and MDA-MB-231 cells following the knockdown or overexpression of *LINC00511* expression (Fig. 4c, d). Some lncRNAs are involved in the regulation of protein synthesis or protein stability in mammalian cells^9^. We found that neither CDKN1B protein synthesis nor stability was affected using the protein synthesis inhibitor cycloheximide (CHX) and the proteasome inhibitor MG132 following the knockdown or overexpression of *LINC00511* expression (Fig. S4a–c). After reviewing the public databases, we aimed to confirm the correlation between *LINC00511* and *CDKN1B* by analysing the RNA-seq data in the CCLE and TCGA databases. The results revealed a negative correlation between *LINC00511* and *CDKN1B* (Fig. 4f, g). The correlation was further validated in 70 breast cancer tissues in the HMUCC cohort (Fig. 4h). The above results suggested that *LINC00511* contributed to breast cancer cell growth by accelerating the G1/S transition in the cell cycle, in part, by regulating *CDKN1B* expression at transcriptional level.

### LINC00511 is required for the epigenetic repression of CDKN1B by interacting with EZH2

**Fig. 5:**
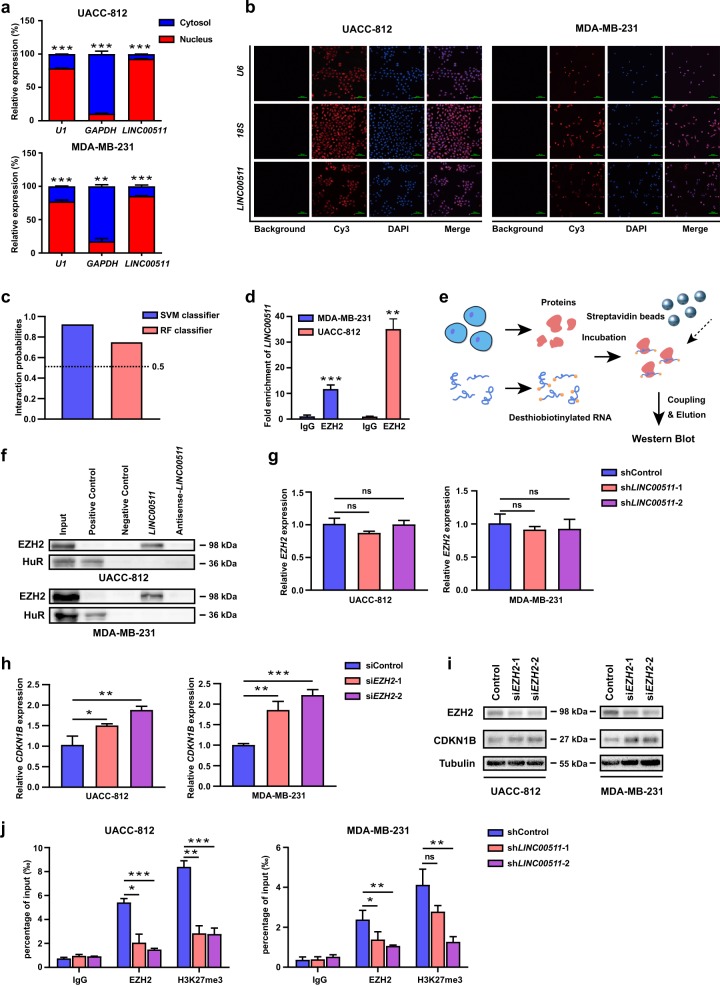
*LINC00511* is required for the epigenetic repression of *CDKN1B* by interacting with EZH2. **a** qRT-PCR analysis of the relative RNA expression levels after nuclear and cytoplasmic RNA separation. *GAPDH* was used as a cytoplasmic marker, and *U1* was used as a nuclear marker. **b** Representative RNA-FISH images of the subcellular location of *LINC00511* in UACC-812 and MDA-MB-231 cells (red). Nuclei were stained with DAPI (blue). *18S rRNA* was used as a cytoplasmic marker, and *U6* was used as a nuclear marker. **c** Analysis of the interaction probabilities of *LINC00511* and EZH2 with the prediction software RPISeq. **d** RNA immunoprecipitation and qRT-PCR analysis of endogenous EZH2 binding to *LINC00511* in UACC-812 and MDA-MB-231 cells with the anti-EZH2 antibody. IgG was used as the control. **e** Schematic of the RNA pull-down assays for the identification of *LINC00511*-associated proteins. **f** Western blot analysis of EZH2 following the pull-down of *LINC00511* or antisense-*LINC00511* in UACC-812 and MDA-MB-231 cells. HuR was used as a positive control. **g** qRT-PCR analysis of the *EZH2* expression of UACC-812 cells and MDA-MB-231 cells following the knockdown of *LINC00511* expression. **h** qRT-PCR analysis of the *CDKN1B* expression of UACC-812 and MDA-MB-231 cells following the knockdown of *EZH2* expression. **i** Western blot analysis of the CDKN1B expression of UACC-812 and MDA-MB-231 cells following the knockdown of *EZH2* expression. **j** ChIP and qRT-PCR analysis of EZH2 and H3K27me3 occupancy at the *CDKN1B* promoter region in UACC-812 and MDA-MB-231 cells following the knockdown of *LINC00511* expression. IgG was used as a negative control. Enrichment was quantified relative to input controls. Data are shown as the mean ± SD. Student’s *t* test was used for the statistical analysis: *p < 0.05; **p < 0.01; ***p < 0.001. Data represent three independent experiments

To explore the specific mechanisms of *LINC00511*, we conducted a fractionated nuclear and cytoplasmic RNA analysis and RNA fluorescence in situ hybridization (RNA-FISH) assays to ensure the subcellular location of *LINC00511* in UACC-812 and MDA-MB-231 cells. *LINC00511* was mainly located in the nucleus (Fig. 5a, b). Previous studies have demonstrated that some lncRNAs in the nucleus recruit the PRC2 complex for epigenetic regulation^19,35^. The methyltransferase EZH2 is the core subunit of the PRC2 complex, and several RNA immunoprecipitation (RIP) experiments have shown a massive number of lncRNAs that bind to EZH2^36^. Thus, we predicted the interaction probabilities between *LINC00511* and EZH2 with the protein-RNA prediction software RPISeq (http://pridb.gdcb.iastate.edu/RPISeq.html) (Fig. 5c). To verify our prediction, we performed RNA immunoprecipitation assays with EZH2 antibodies. The results revealed that *LINC00511* could bind to EZH2 in UACC-812 and MDA-MB-231 cells (Fig. 5d). As shown in Fig. 5e, RNA pull-down assays also confirmed that *LINC00511* could directly interact with EZH2 in UACC-812 and MDA-MB-231 cells (Fig. 5f).

Subsequently, we wanted to determine whether EZH2 was involved in the repression of *CDKN1B*. We found no effect on EZH2 expression following the knockdown of *LINC00511* expression in UACC-812 and MDA-MB-231 cells (Fig. 5g). Simultaneously, the knockdown efficiency of *EZH2* expression in UACC-812 and MDA-MB-231 cells was shown in Fig. S5a, b. qRT-PCR and western blot analyses confirmed that the expression of *CKDN1B* was increased following the knockdown of *EZH2* expression in UACC-812 and MDA-MB-231 cells (Fig. 5h, i). So far, we hypothesized that *LINC00511* regulated *CDKN1B* expression through EZH2-mediated H3K27me3 trimethylation at the promoter region of *CDKN1B*. As shown in Fig. 5j, ChIP assays were performed to confirm that the occupancy capacity of EZH2 and H3K27me3 at the specific promoter region of *CDKN1B* was impaired after knocking down *LINC00511* in UACC-812 and MDA-MB-231 cells.

### Knockdown of LINC00511 expression represses tumour growth in vivo

**Fig. 6:**
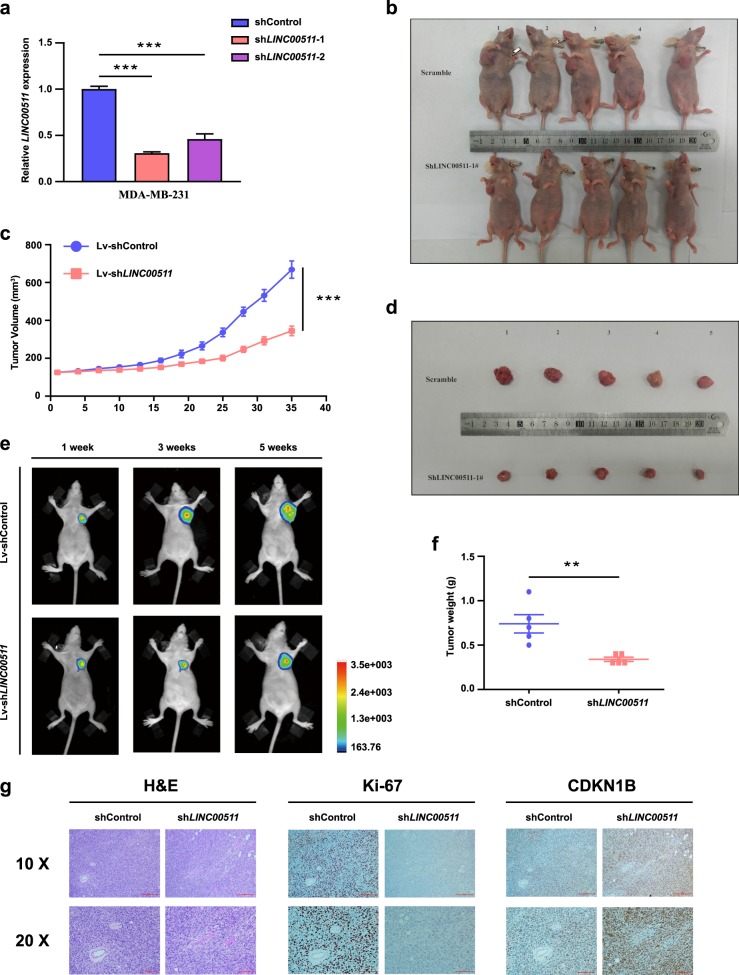
Knockdown of *LINC00511* expression represses tumour growth in vivo. **a** qRT-PCR analysis of the knockdown efficiency of *LINC00511* expression in luciferase-labelled MDA-MB-231 cells. **b** Representative images of nude mice bearing tumours from the shcontrol and sh*LINC00511* cohorts. **c** The growth curves of tumour volumes calculated every 3 days after injection in the shcontrol and sh*LINC00511* cohorts. **d** Representative images of tumours from the shcontrol and sh*LINC00511* cohorts. **e** Representative bioluminescent images of nude mice bearing tumours of luciferase-labelled MDA-MB-231 cells from the shcontrol and sh*LINC00511* cohorts. Image capture occurred during the 1st, 3rd and 5th weeks. **f** Scatter diagram depiction of the final tumour weights in shcontrol and sh*LINC00511* cohorts. **g** Representative images of haematoxylin and eosin (H&E) staining and Ki-67 and CDKN1B immunostaining of tumours from the shcontrol and sh*LINC00511* cohorts. Data are shown as the mean ± SD. Student’s *t* test was used for the statistical analysis: *p < 0.05; **p < 0.01; ***p < 0.001. Data represent three independent experiments

For the purpose of further confirming that *LINC00511* affected tumour growth in vivo, luciferase-labelled MDA-MB-231 cells transfected with a shcontrol vector or a short hairpin RNA (shRNA) against *LINC00511* (sh*LINC00511*) were injected into nude mice. We chose luciferase-labelled MDA-MB-231 cells transfected with sh*LINC00511–1* as the experimental cohort because of the higher knockdown efficiency of *LINC00511* expression in MDA-MB-231 cells, as shown in Fig. 6a. On the 35th day following injection, the tumours that developed in the sh*LINC00511* cohort were remarkably smaller than those that developed in the shcontrol cohort (Fig. 6b and d). Moreover, the average tumour volumes and respective weights were remarkably lower in the sh*LINC00511* cohort than the shcontrol cohort (Fig. 6c and f). Bioluminescent imaging was utilized to detect tumour growth dynamically every other week. As shown in Fig. 6e, the xenograft growth ability of MDA-MB-231 cells in the sh*LINC00511* cohort was weaker than that in the shcontrol cohort.

Moreover, the microscopic observation of tumours revealed that tumours that developed in the shcontrol cohort showed stronger Ki-67 expression than those in the sh*LINC00511* cohort, and the tumours that developed in the sh*LINC00511* cohort showed stronger CDKN1B expression than those that developed in the shcontrol cohort, as detected by immunohistochemistry (IHC) analysis (Fig. 6g). These results supported a role for *LINC00511* in promoting breast cancer tumour growth in vivo.

## Discussion

With the large population of breast cancer patients worldwide, the heterogeneity of breast cancer is still an obstacle in the assessment and treatment of breast cancer. The emergence of an evaluation system for molecular classification promoted the development of tailored individualized therapy^37,38^. Among the major molecular receptors, ER status separated breast cancer into distinct subgroups of which 70% express ER^39^. While ER, which is regarded as the decisive hormone receptor in breast cancer, is essential in the algorithm for treatment decision making, a poor prognosis can be observed in ER-negative patients^40^. Further investigation and study of the mechanisms through which ER-negative breast cancers become aggressive and eventually evade traditional therapy is of clinical importance.

**Fig. 7:**
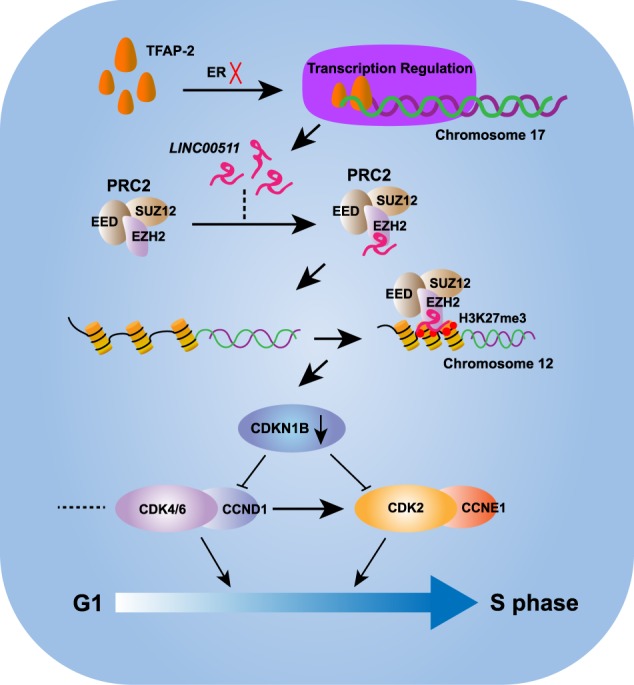
Schematic illustration of the possible regulatory mechanisms in this study. ER deficiency promoted TFAP-2-inducible *LINC00511* expression by enhancing the occupancy efficiency of TFAP-2 at specific promoter regions. The ER-negative-associated *LINC00511*, which was mainly located in the nucleus, repressed the expression of *CDKN1B* via interacting with EZH2 to recruit PRC2 to mediate histone methylation, contributing to the G1/S transition to sustain cell proliferation

In previous studies of ER-positive breast cancer, the activation of ER signalling was found to be involved in crosstalk with multiple signalling pathways, such as PI3K/AKT/mTOR or ER-CCND1-CDK4/6-RB, and to promote cell cycle progression^41–43^. Compared with ER-positive breast cancer, the specific oncogenic molecular and regulatory mechanisms remain poorly understood in ER-negative breast cancer. In this study, we identified several potential ER-negative-associated lncRNAs in breast cancer and mechanistically elaborated the oncogenic function of the one of the most intriguing candidates in ER-negative breast cancer. We firmly believed that the functional interpretation of other candidates will contribute to our understanding of ER-negative biology. lncRNAs function through multiple mechanisms, such as participating in epigenetic regulation, endogenous competition regulation and endogenous transport regulation^44–46^. According to the prioritization of variation in ER-negative-associated lncRNAs, we identified and investigated the oncogenic function of *LINC00511* in ER-negative breast cancer. At the transcriptional level, ER deficiency directly affected the expression of *LINC00511* activated by TFAP-2 in breast cancer cells. We determined that *LINC00511* promotes tumour growth and inhibits apoptosis. The study of the interaction between *LINC00511* and EZH2, the catalytic subunit of PRC2, was a crucial step towards understanding the mechanisms through which *LINC00511* exerted its oncogenic function in breast cancer. Through the epigenetic silencing of *CDKN1B*, which acts as a brake to halt cell cycle progression, *LINC00511* accelerated the G1/S transition to sustain cell proliferation (Fig. 7). Further investigation of *LINC00511* in oncogenesis and cancer progression is necessary.

Along with the deeper understanding of molecule-mediated oncogenic mechanisms in ER-negative breast cancer, we expect improvements in treatment strategies in ER-negative breast cancer. It is necessary to prolong survival and alter the poor prognosis of ER-negative breast cancer patients. In recent years, small molecule compound drugs targeting novel tumour mediators have brought us hope, but the treatment of ER-negative breast cancer remains a great clinical challenge^47–49^. Ultimately, our study provides insight into the oncogenic function of *LINC00511* in promoting ER-negative tumourigenesis, and further investigation of other candidates is likely to yield a greater understanding of ER-negative breast cancer biology. Moreover, these lncRNAs may be exploited as potential anticancer treatments in the future.

## Materials and methods

### Cell lines, cell culture and treatment

All the breast cancer cell lines were obtained from the Institute of Biochemistry and Cell Biology of the Chinese Academy of Sciences (Shanghai, China) and periodically authenticated (Cellbio). Unless otherwise specified, cells were maintained in Roswell Park Memorial Institute (RPMI) 1640 medium (Gibco, Carlsbad, CA, USA), Dulbecco’s modified Eagle’s medium (DMEM) plus GlutaMAX (Gibco, Carlsbad, CA, USA) or Leibovitz’s L15 medium (PYG0038, Boster, China) supplemented with 10% foetal bovine serum (FBS; 0500, ScienCell, USA) and 1% penicillin-streptomycin in a humidified incubator at 37 °C with 5% CO_2_ or air. For the ER deprivation experiments, MCF7 cells were treated with the ER antagonist tamoxifen (T5648–1G, Sigma, USA), for 24 h. For the ubiquitination assay, UACC-812 and MDA-MB-231 cells were treated with MG132 (HY-13259, MCE, USA) for 40 min. For the CHX chase assay, MDA-MB-231 cells were incubated with 50 μg/ml CHX (HY-12320, MCE, USA) for the indicated durations (0, 15, 30, 60, 120 and 180 min), as previously described^9^.

### Knockdown and overexpression studies

The stable knockdown of *LINC00511* and *ESR1* was accomplished by lentiviral constructs containing two different *LINC00511* and *ESR1* shRNAs or no targeting shRNA (Umibio (Shanghai) Co.,Ltd, China) in the presence of polybrene (107689, Sigma). The transduced cells were cultured in culture media containing 1 µg ml^−1^ puromycin (Catalogue Number 540411, Calbiochem, USA) for 2 weeks. The knockdown of *TFAP-2* and *EZH2* was accomplished with a small interfering RNA (siRNA; HANBIO, China). The transfections were performed with INTERFERin® (Polyplus-transfection® SA) according to the manufacturer’s instructions. The target sequences used for the shRNAs or siRNAs are listed in Table S3.

The overexpression of *LINC00511* was accomplished with a plasmid containing full-length *LINC00511* cloned into the pcDNA3.1 vector between theEcoRI and KpnI sites (Umibio (Shanghai) Co.,Ltd, China). The transfections were performed with jetPRIME® (Polyplus-transfection® SA) according to the manufacturer’s instructions. Cells were collected at 48 h post transfection.

The knockdown and overexpressing cell lines were identified by a qRT-PCR assay or western blot analysis.

### qRT-PCR assay

The E.Z.N.A.® Total RNA Kit I (Catalogue Number R6834–01, Omega Bio-Tek, USA) was utilized to isolate RNA from cell lysates. From 1 µg of isolated RNA, a Transcriptor First Strand cDNA Synthesis Kit and Random Primer (Catalogue Number 04897030001, Roche, USA) were used to generate cDNA according to the manufacturer’s protocol. The 7500 Fast Real-Time PCR system (Applied Biosystems, USA) was utilized for qRT-PCR. A relative quantification method was used to analyse the qRT-PCR data, and actin was used as a reference for the mRNAs or lncRNAs. Each sample was analysed in triplicate. The primer sequences synthesized by Shanghai Generay Biotech Co.,Ltd, are listed in Table S3.

### Western blot assay and antibodies

Western blot assays were performed by running cell lysates on 8–10% SDS polyacrylamide gels (Solarbio) to separate proteins. The separated proteins were then transferred to a polyvinylidene fluoride membrane via wet transfer at 300 mA for 60–90 min. After incubating with blocking buffer (Becton Dickinson, USA) for 1 h, the indicated antibodies were added to the membrane and incubated at 4 °C overnight. The blots were incubated with goat anti-rabbit IgG H&L (HRP) or goat anti-mouse IgG H&L (HRP) for 1 h at room temperature. FluorChem HD2 (Protein Sample, USA) was used to detect the proteins via enhanced chemiluminescence. All antibodies used in this study are described in Table S4.

### Subcellular fractionation

Cellular fractionation was performed using NE-PER^TM^ Nuclear and Cytoplasmic Extraction Reagents (Catalogue Number 78835, Thermo Fisher) according to the manufacturer’s instructions. qRT-PCR was performed to detect the isolated RNA, with GAPDH and U1 used as the reference for cytoplasmic and nuclear RNA, respectively.

### Fluorescence in situ hybridization

RNA-FISH was performed with a Ribo^TM^ Fluorescence In Situ Hybridization Immobilized Kit (Catalogue Number 10910, RiboBio Co., Ltd, China) according to the manufacturer’s instructions. *LINC00511*, U6 and 18S (referencesfor the nucleus and cytoplasm) hybridized with cy3 oligonucleotide probes were observed with a confocal laser scanning microscope (FV1200, Olympus, Japan).

### Chromatin immunoprecipitation (ChIP)

An EZ-ChIP^TM^ ChIP Kit (Catalogue Number #17–371, Millipore, USA) was utilized to perform ChIP assays according to the manufacturer’s instructions. Briefly, ∼2 × 10^7^ shcontrol MCF7 cells or MCF7 cells in which the stable knockdown of ER was validated were used for each ChIP assay. Cells were crosslinked using 1% formaldehyde for 20 min, and cross-linking was quenched for 10 min at room temperature using a 1/10 volume of 1.25 M glycine. The cells treated with enzyme lysis and sonication yielded an average chromatin fragment size of 300–500 bp. The DNAs bound to the antibody against TFAP-2 (sc-12762, Santa Cruz, USA) via overnight incubation at 4 °C were purified with a Universal DNA Purification Kit (DP214, Tiangen, China) according to the manufacturer’s instructions. ChIP DNA was subjected to quantitative PCR and is reported as % input ± standard error of the mean (S.E.M.). The primers used for PCR are listed in Table S3.

### RNA immunoprecipitation (RIP)

Magna RIP^TM^ RNA-Binding Protein Immunoprecipitation Kits (Catalogue Number #17–700, Millipore, USA) were used according to the manufacturer’s instructions. The abundance of *LINC00511* was detected by quantitative PCR using total RNA as an input control. The antibody used for RIP is listed in Table S4.

### RNA pull-down assay

The T7 RiboMAX^TM^ Express Large Scale RNA Production System (Catalogue Number P1320, Promega, USA) was used to produce abundant *LINC00511*, including sense and antisense RNAs, in vitro according to the manufacturer’s instructions. The Pierce™ RNA 3′ End Desthiobiotinylation Kit (Catalogue Number 20163, Thermo Fisher, USA) was applied to label the 3′ ends of the *LINC00511* RNAs (including sense and antisense) with a desthiobiotin tag according to the manufacturer’s instructions. Protein-RNA interactions were determined using a Pierce™ Magnetic RNA-Protein Pull-Down Kit (Catalogue Number 20164, Thermo Fisher, USA) with lysates from MDA-MB-231 and UACC-812 cells. Then, western blot assays were used to detect the precipitated proteins.

### Cell proliferation and colony formation assays

MDA-MB-231 and UACC-812 cell proliferation and colony formation abilities were assessed as previously described^9^.

### EdU proliferation assay

A Cell-Light^TM^ EdU Apollo567 In Vitro Kit (Catalogue Number C10310–1, RiboBio, China) was used to perform the EdU proliferation assay according to the manufacturer’s instructions as previously described^50^.

### Apoptosis and cell cycle analysis

MDA-MB-231 and UACC-812 cells expressing the indicated constructs were treated with the Cell Cycle Staining Kit (Catalogue Number 70-CCS012, Multiscience, China) and an Annexin V, FITC Apoptosis Detection Kit (Catalogue Number AD10, Dojindo, Japan) according to the manufacturer’s instructions and then analysed by flow cytometry (BD FACSCalibur, USA). The results are presented as the percentage of cells in each phase.

### Xenograft analysis

All experimental procedures were approved by the Institutional Animal Care and Use Committee of the Center of Harbin Medical University and conformed to all regulatory standards. A total of 5 × 10^6^ luciferase-labelled MDA-MB-231 control cells or *shLINC00511–1*cells suspended in 0.2 ml of PBS with phenol-red-free Matrigel (Catalogue Number 356234, Corning, USA) (1:1) were injected into the axilla of 5-week-old pathogen-free female athymic BALB/c mice obtained from Shanghai SLAC Laboratory Animal Co., Ltd (www.slaccas.com, China). Bioluminescence imaging was carried out using a Carestream Image Station System (Multimodal Pro Light Source, Carestream Health, Inc., CA). When the tumours became palpable, tumour volume was assessed by digital calliper measurements using the formula (width^2^ × length)/2 (mm^3^), and the whole body weight was measured once every 3 days. All mice were euthanized at the end of the experiment.

### IHC assay

The tumours were stained for H&E and immunostained for Ki-67 and CDKN1B as previously described^9^.

### Statistical analysis

Statistical analysis was performed using SPSS 17.0 software (SPSS Software, USA) and GraphPad Prism 8 (GraphPad Software, USA). Student’s *t* test and the chi-square test were used to determine significant differences where appropriate. Survival was calculated by the Kaplan–Meier method, with the log-rank test applied for comparison. All statistical tests were two-sided, and a probability level of 0.05 indicated statistical significance.

## Supplementary information

Supplementary legends.

Figure S1

Figure S2

Figure S3

Figure S4

Figure S5

Table S1

Table S2

Table S3

Table S4
