# Supplementary material for: The transcriptional landscape of lncRNAs reveals the oncogenic function of LINC00511 in ER-negative breast cancer
Source: Cell Death Dis. 2019 Aug 8;10(8):599. doi: 10.1038/s41419-019-1835-3 (PMC6687715; doi:10.1038/s41419-019-1835-3)
Supplement: Supplementary file 1 — Supplementary legends. [file 41419_2019_1835_MOESM1_ESM.docx]

**Figure S1.** Characteristics of *LINC00511* in protein-coding potential.

1. Protein-coding potential analysis of *LINC00511* using the CPAT database.
2. Protein-coding potential analysis of *LINC00511* using the CPC database.
3. Western blot analysis of ribosomal protein LP0 following the pull-down of *LINC00511* or antisense-*LINC00511* in UACC-812 cells.

Data are shown as the mean ± SD. Student’s t test was used for the statistical analysis: **p*<0.05; ***p*< 0.01; ****p*< 0.001. Data represent three independent experiments.

**Figure S2.** Expression levels of *LINC00511* in breast cancer cells.

1. qRT-PCR analysis of *LINC00511* expression in 9 breast cancer cells.

Data are shown as the mean ± SD. Student’s t test was used for the statistical analysis: **p*<0.05; ***p*< 0.01; ****p*< 0.001. Data represent three independent experiments.

**Figure S3.** Validation of *LINC00511* expression in treated breast cancer cells.

1. qRT-PCR analysis of the knockdown efficiency of *LINC00511* expression in UACC-812 cells.
2. qRT-PCR analysis of the knockdown efficiency of *LINC00511* expression in MDA-MB-231 cells.
3. qRT-PCR analysis of the overexpression efficiency of *LINC00511* in MDA-MB-231 cells.

Data are shown as the mean ± SD. Student’s t test was used for the statistical analysis: **p*<0.05; ***p*< 0.01; ****p*< 0.001. Data represent three independent experiments.

**Figure S4.** *LINC00511* regulates *CDKN1B* expression at the transcriptional level.

1. Western blot analysis of CDKN1B expression in MDA-MB-231 cells following the knockdown of *LINC00511* expression with CHX treatment.
2. Western blot analysis of CDKN1B expression in UACC-812 and MDA-MB-231 cells following the knockdown of *LINC00511* expression with MG132 treatment.
3. Western blot analysis of CDKN1B expression in MDA-MB-231 cells following the overexpression of *LINC00511*with MG132 treatment.

Data are shown as the mean ± SD. Student’s t test was used for the statistical analysis: **p*<0.05; ***p*< 0.01; ****p*< 0.001. Data represent three independent experiments.

**Figure S5.** Validation of *EZH2* expression in treated breast cancer cells.

1. qRT-PCR analysis of the knockdown efficiency of *EZH2* expression in UACC-812 cells.
2. qRT-PCR analysis of the knockdown efficiency of *EZH2* expression in MDA-MB-231 cells.

Data are shown as the mean ± SD. Student’s t test was used for the statistical analysis: **p*<0.05; ***p*< 0.01; ****p*< 0.001. Data represent three independent experiments.
