# Supplementary figures and images for: The transcriptional landscape of lncRNAs reveals the oncogenic function of LINC00511 in ER-negative breast cancer
Source: Cell Death Dis. 2019 Aug 8;10(8):599. doi: 10.1038/s41419-019-1835-3 (PMC6687715; doi:10.1038/s41419-019-1835-3)

**a**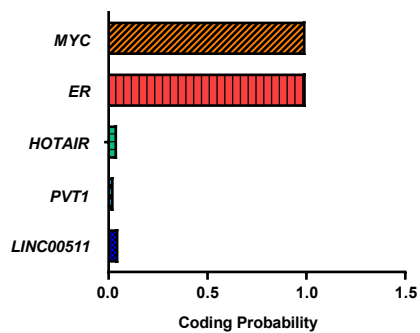**b**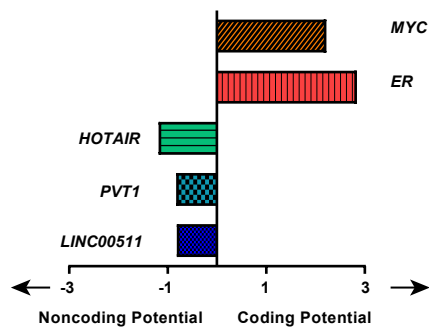**c**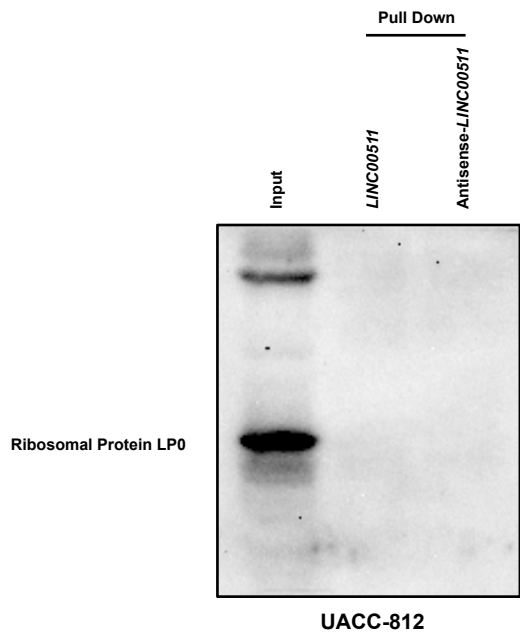

Supplement: Supplementary file 2 — Figure S1 [file 41419_2019_1835_MOESM2_ESM.pdf]

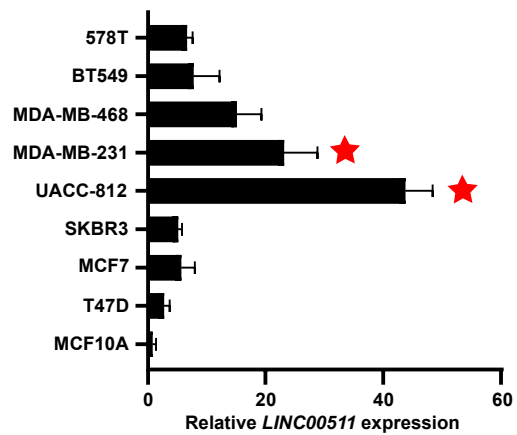

Supplement: Supplementary file 3 — Figure S2 [file 41419_2019_1835_MOESM3_ESM.pdf]

**a**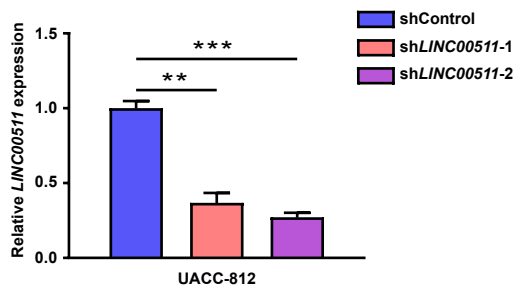**b**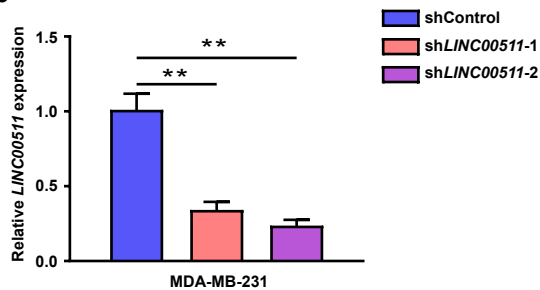**c**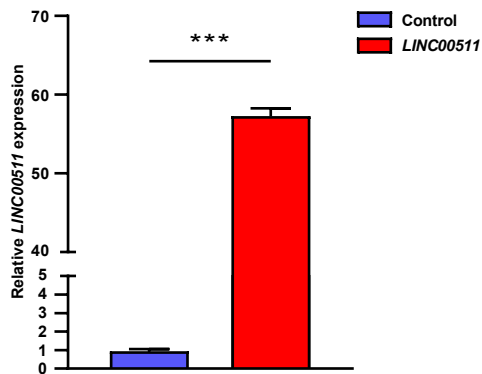

Supplement: Supplementary file 4 — Figure S3 [file 41419_2019_1835_MOESM4_ESM.pdf]

a

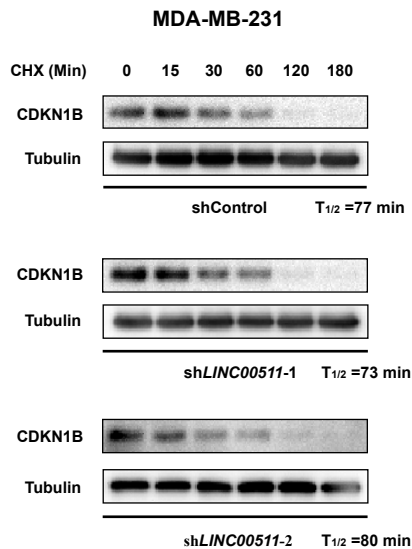

b

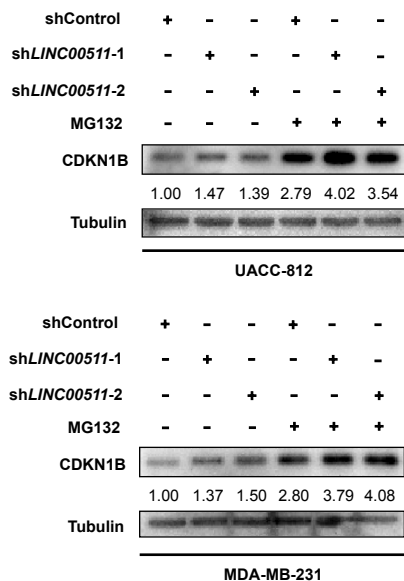

c

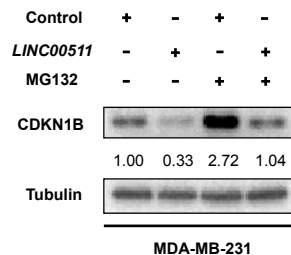

Supplement: Supplementary file 5 — Figure S4 [file 41419_2019_1835_MOESM5_ESM.pdf]

**a**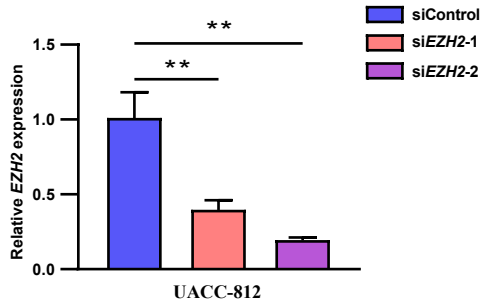**b**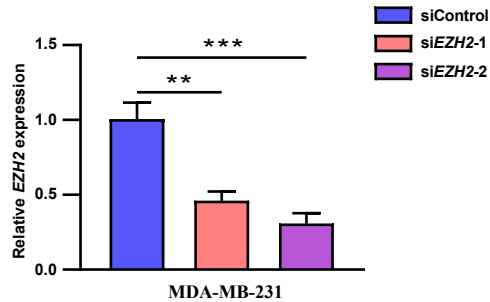

Supplement: Supplementary file 6 — Figure S5 [file 41419_2019_1835_MOESM6_ESM.pdf]
