# Supplementary material for: The transcriptional landscape of lncRNAs reveals the oncogenic function of LINC00511 in ER-negative breast cancer
Source: Cell Death Dis. 2019 Aug 8;10(8):599. doi: 10.1038/s41419-019-1835-3 (PMC6687715; doi:10.1038/s41419-019-1835-3)
Supplement: Supplementary file 7 — Table S1 [file 41419_2019_1835_MOESM7_ESM.docx]

| **15** up-regulated lncRNAs |  |
| --- | --- |
| Cancer versus Normal ER- versus ER+  Gene name Ensembl ID Genomic Coordinates  Fold change *p* Value Fold change *p* Value | |
| *LINC00511*  ENSG00000227036 chr17:72598041-72640472(-) 7.06 4.54E-42 5.79 1.19E-22  *U62317.1* ENSG00000272666 - 7.54 4.04E-41 3.76 1.24E-11  *LINC01096* ENSG00000246095 chr4:13546076-13547824(-) 4.99 1.77E-28 3.69 1.89E-09  *LINC02086* ENSG00000244649 chr17:48655662-48707346(+) 12.96 8.26E-28 3.64 2.07E-06  *LINC01842*  ENSG00000267147 chr19:14333743-14343913(+) 9.63 1.42E-44　　　　　3.24 5.33E-13  *LINC01833* ENSG00000259439 chr2:44921080-44939199(-) 7.52 7.03E-31 3.13 2.02E-09  *LncUSMycn*  ENSG00000223850 chr2: 15920399-15936018(+) 14.97 4.99E-28 2.98 1.55E-05  *C10orf91* ENSG00000180066 chr10: 132445189- 132448306(+) 4.08 7.62E-24 2.77 1.77E-05  *LINC02195* ENSG00000236481 chr16: 26584799- 26595452(-) 5.56 9.32E-42 2.44 6.42E-09  *AL022316.1* ENSG00000230107 - 5.19 3.23E-40 2.34 3.81E-07  *LINC02416* ENSG00000257924 chr12: 47353719- 47370082(+) 5.15 9.44E-26 2.31 4.77E-09  *AP000251.1* ENSG00000237594 - 5.58 3.6E-87 2.29 9.81E-15  *AC138904.1*  ENSG00000246465 - 5.70 1.15E-64 2.23 5.8E-09  *LINC01615* ENSG00000223485 chr6: 169158092- 169162989(-) 3.84 3.46E-39 2.17 1.62E-05  *LINC00337* ENSG00000225077 chr1: 6236240- 6239444(+) 6.74 6.46E-90 2.13 5.43E-12 | |
